# Supplementary material for: Achieving Discharge Limits in Single-Stage Domestic Wastewater Treatment by Combining Urban Waste Sources and Phototrophic Mixed Cultures
Source: Microorganisms. 2023 Sep 15;11(9):2324. doi: 10.3390/microorganisms11092324 (PMC10536668; doi:10.3390/microorganisms11092324)
Supplement: Supplementary file 1 [file microorganisms-11-02324-s001.zip › microorganisms-2557098-supplementary.pdf]

SUPPLEMENTARY INFORMATION

S1. <u>Classification of microbial evolution when genera are greater than 1% relative abundance</u>

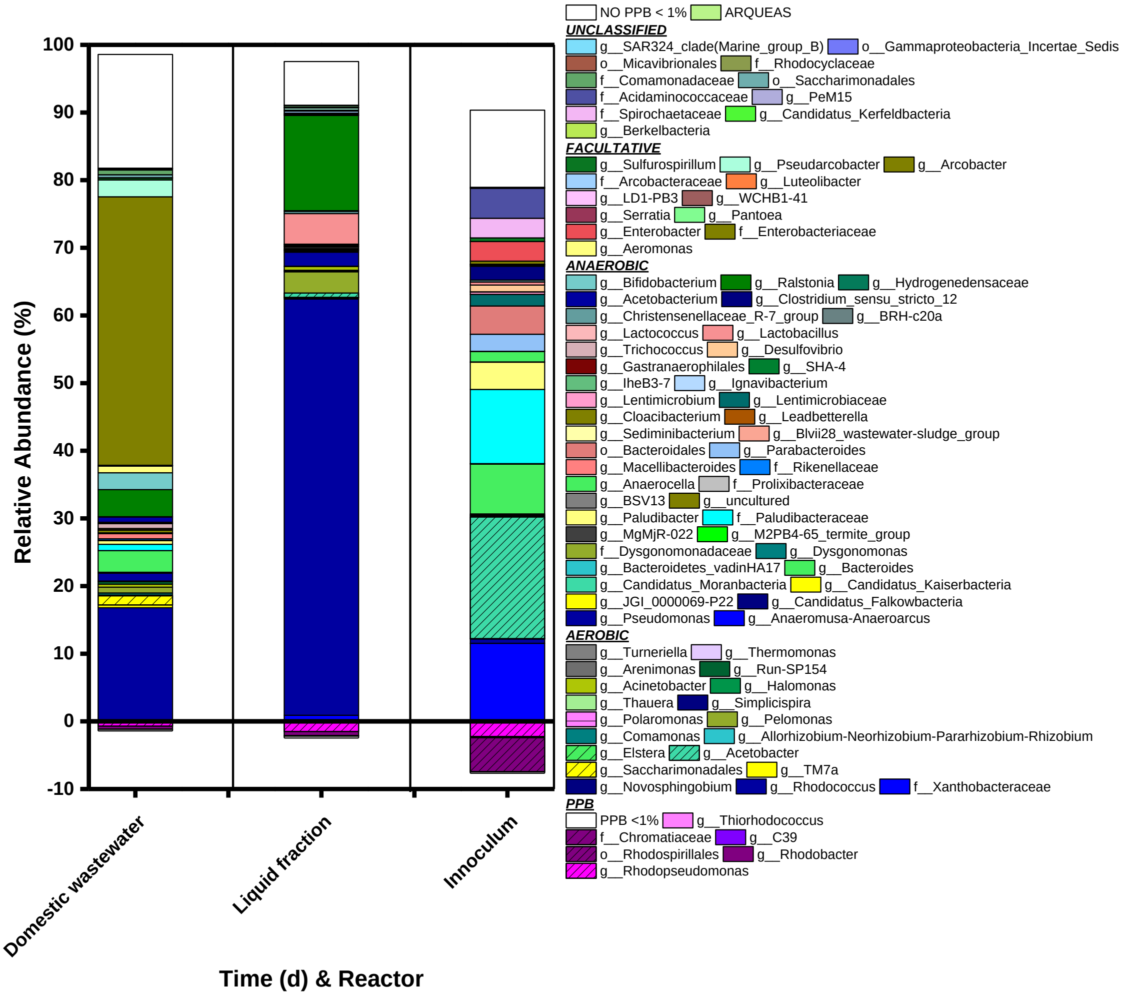

Figure S1. Qualification of the microbial population taking into account all genera >1% in the wastewater, the liquid fraction and the inoculum.

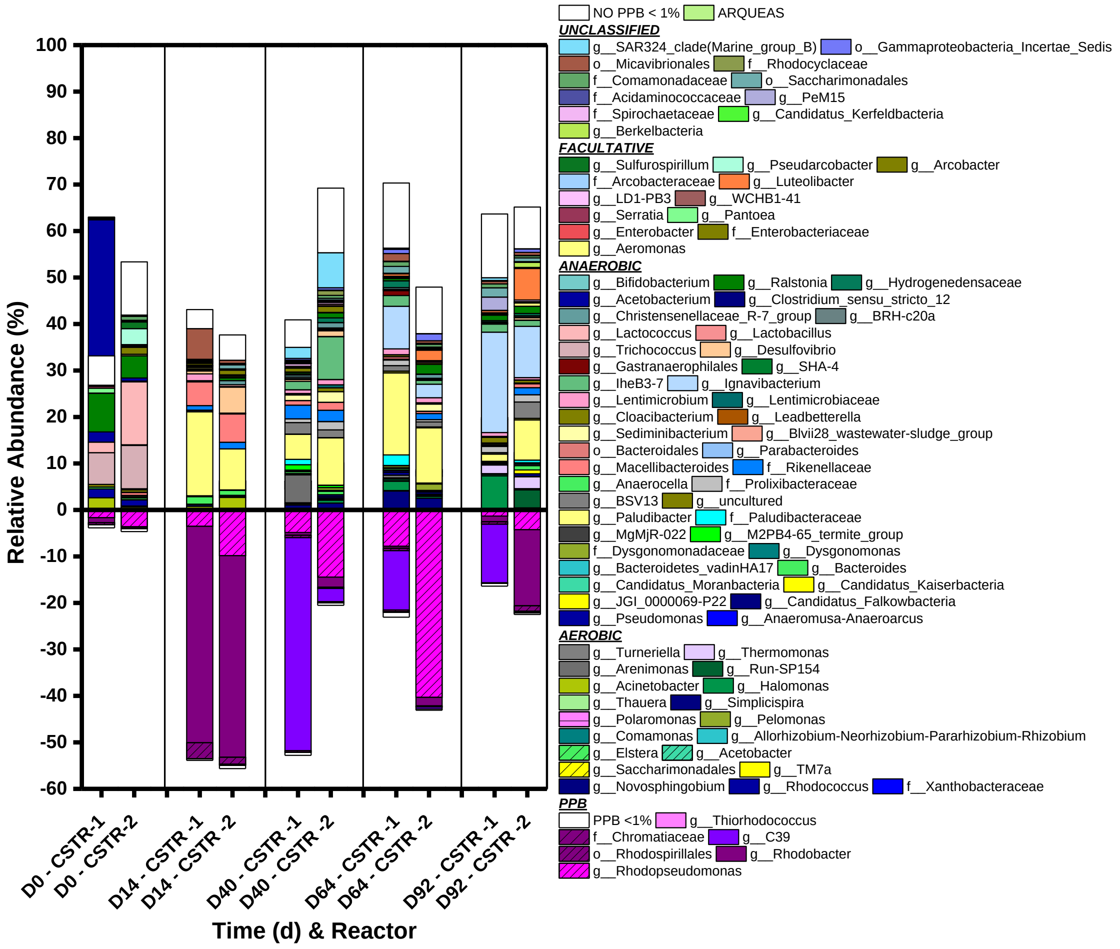

Figure S2. Evolution of the microbial population considering all genera >1% during CSTR-mode.

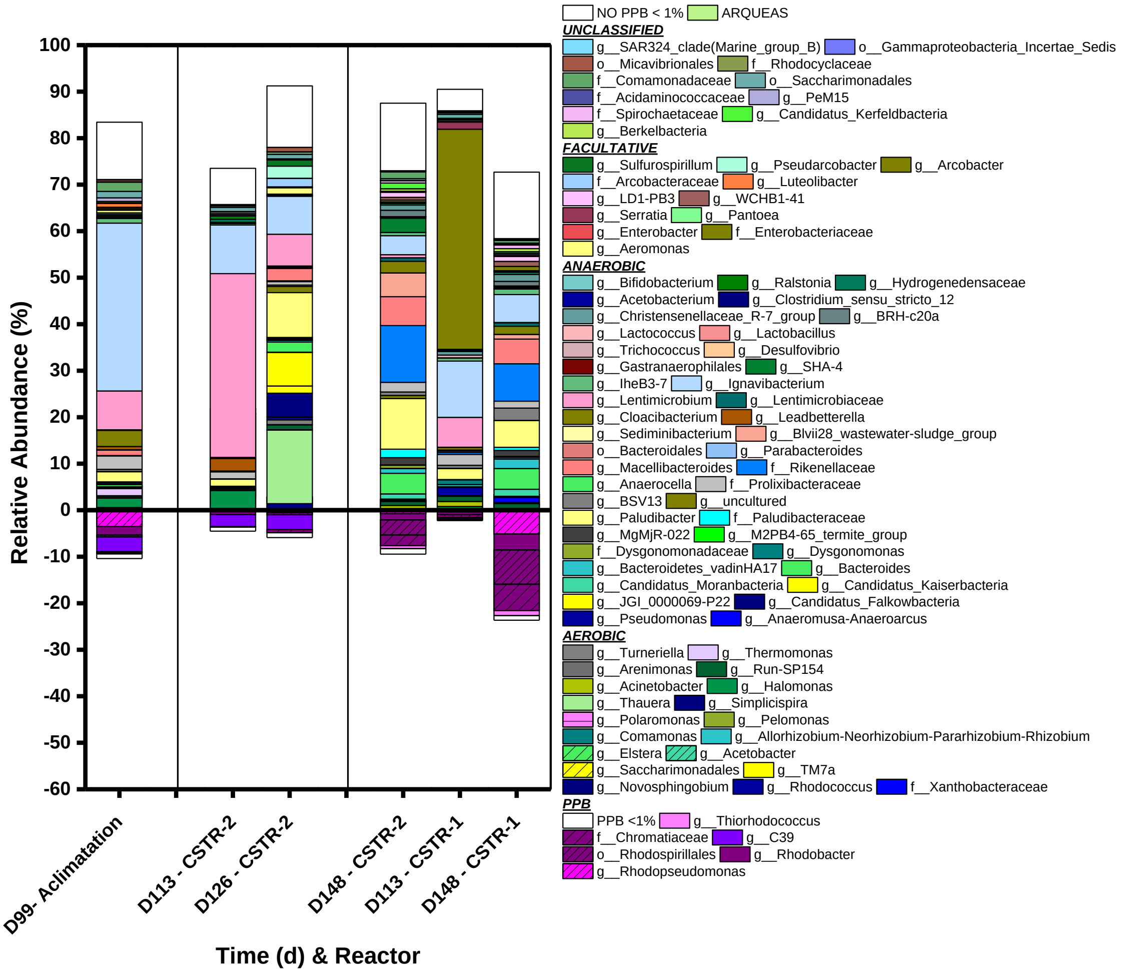

Figure S3. Evolution of the microbial population taking into account all genera >1% during MBR-mode.

S2. Description of the microbial community analysis by Illumina MiSeq.

The quantification, amplification (PCR), 16S rRNA gene measurements, and the amplicon taxonomic annotation and comparative analysis were outsourced to Instituto ISABIAL-FISABIO, Hospital General Universitario de Alicante, Alicante, Spain. 16S rDNA gene amplicons were obtained following the 16S rDNA gene Metagenomic Sequencing Library Preparation Illumina protocol (Cod. 15044223 Rev. A). The gene-specific sequences used in this protocol target the 16S rDNA gene V3 and V4 region. Illumina adapter overhang nucleotide sequences are added to the gene-specific sequences. The primers are selected from the Klindworth et al. (2013). The full length primer sequences, using standard IUPAC nucleotide nomenclature, to follow the protocol targeting this region are:

- 16S rDNA gene Amplicon PCR Forward Primer = 5' (TCGTCGGCAGCGTCAGATGTGTATAAGAGACAGCCTACGGGNGGCWGCAG)
- 16S rDNA gene Amplicon PCR Reverse Primer = 5' (GTCTCGTGGGCTCGGAGATGTGTATAAGAGACAGGACTACHVGGGTATCTAATCC).

We used microbial genomic DNA (5 ng/μl in 10 mM Tris pH 8.5) to initiate the protocol. After 16S rDNA gene amplification, the mutiplexing step was performed using using Nextera XT Index Kit (FC-131-1096). We run 1 μl of the PCR product on a Bioanalyzer DNA 1000 chip to verify the size, the expected size on a Bioanalyzer trace is ~550 bp. After size verification the libraries were sequenced using a 2x300pb paired-end run (MiSeq Reagent kit v3 (MS-102-3001)) on a MiSeq Sequencer according to manufacturer's instructions (Illumina). Quality assessment was performed by the use of prinseq-lite program (Schmieder, et al., 2011) applying following parameters: min_length: 50, trim_qual_right: 30, trim_qual_type: mean, trim_qual_window: 20. Data have been obtained using an ad-hoc pipeline written in RStatistics environment (R Core Team, 2012), making use of several Open Source libraries such as gdata , vegan , etc. Data have been grouped and stratified according to the metadata file provided by the user. The sequence data have been analyzed using qiime2 pipeline originally cited in Caporaso, et al. (2010). Metataxonomy analysis was performed using some of qiime2 plugins. Denoising, paired-ends joining, and chimera depletion was performed starting from paired ends data using DADA2 pipeline (Callahan, et al., 2016). Taxonomic affiliations have been assigned using the Naive Bayesian classifier integrated in quiime2 plugins. Database used for this taxonomic assignation was the SILVA_release_132 (Quast, et al., 2013). Comparative data analysis and plot generation considered only the major contributing functional groups (>1%).

References

R. Schmieder et al. "Quality control and preprocessing of metagenomic datasets". In: *Bioinformatics* 27.6 (mar. 2011). [PubMed Central:PMC3051327] [DOI:10.1093/bioinformatics/btr026] [PubMed:21278185] , pp. 863-864.

R Core Team. *R: A Language and Environment for Statistical Computing*. ISBN 3-900051-07-0. R Foundation for Statistical Computing. Vienna, Austria, 2012. <URL: http://www.R-project.org/>.

J. G. Caporaso et al. "QIIME allows analysis of high-throughput community sequencing data". In: *Nat. Methods* 7.5 (may. 2010). [PubMed Central:PMC3156573] [DOI:10.1038/nmeth.f.303] [PubMed:20383131] , pp. 335-336.

B. J. Callahan et al. "DADA2: High-resolution sample inference from Illumina amplicon data". In: *Nat. Methods* 13.7 (jul. 2016). [PubMed Central:PMC4927377] [DOI:10.1038/nmeth.3869] [PubMed:27214047] , pp. 581-583.

C. Quast et al. "The SILVA ribosomal RNA gene database project: improved data processing and web-based tools". In: *Nucleic Acids Res.* 41.Database issue (ene. 2013). [PubMed Central:PMC3531112][DOI:10.1093/nar/gks1219] [PubMed:23193283] , pp. D590-596.
